# The State Association of Health Officers of Texas

**Published:** 1901-05

**Authors:** Wm. Myers

**Affiliations:** County Health Officer Guadalupe County, Seguin, Texas


### Society Notes.

### THE STATE ASSOCIATION OF HEALTH OFFICERS OF TEXAS.

### A Brief History of the Recent Outbreak of Smallpox in Guadalupe County, and Remarks.

WM. MYERS, M. D., COUNTY HEALTH OFFICER GUADALUPE COUNTY, SEGUIN, TEXAS.

[Meeting of March 21, 1901—continued.]

On November 10th I was called fifteen miles northeast of Seguin to investigate a suspected case of smallpox. I found at the place a colored woman in bed, whose exposed parts of face, hands and arms showed a fully developed eruption of smallpox. I also found in the same room the corpse of a six months old baby, who had died of the smallpox a few hours previously. The house was occupied by a negro family consisting of two adults and four children in addition to the sick one. This house was immediately placed under quarantine, as well as another house in close proximity, the inmates of which had been in communication with the one in which the disease had made its appearance. This latter family, consisting of three adults and five children, and those in the other house were vaccinated the following day, but without any result as to preventing the disease, as all of them subsequently developed cases of smallpox. It was learned that a number of negroes returning from a funeral the preceding day had called at these houses. They lived within a radius of five to seven miles, and they were put under observation by a patrol, which was established within a few days, as soon as necessary guards could be obtained. By these means within about two weeks cases were discovered in eight different families of negroes, wihch were removed to the locality where the first case had appeared, and where, by that time, already eight or ten cases existed. They were accommodated in tents, and nurses, medicines and provisions furnished them by the county. The inmates of the houses from which patients were removed were vaccinated under compulsion and detained three weeks. A case of smallpox was discovered in Kingsbury in December, and was also removed to the same camp, and the whole family, in which subsequently five other cases developed, placed in detention. A few days later a white boy, living five miles from the nearest place at which smallpox had made its appearance, rode into Kingsbury for treatment with a well-marked eruption on his face. He was sent home and the house was quarantined the following day. The other inmates of the house refused vaccination and subsequently were attacked, and the quarantine on their premises could not be raised until February 1st. A married sister of the white boy first attacked, living about three miles distant, who had been on a visit to her mother's on the day her brother went to Kingsbury, was attacked within a week after her visit, also her husband and two children, all of whom had not been vaccinated. This family was already under quarantine when the first case developed. From November 10th to January 29, 1901, when the smallpox camp on York's creek was broken up, there were seventy-seven cases of smallpox in this section of the county, of which eight were whites and sixty-nine negroes. Among the latter were three deaths, one adult and two infants. A negro woman, while in the second stage of the disease, gave birth to a child, which developed smallpox when a week old; this infant recovered.

On January 5th, 1901, it was reported that smallpox existed in a colored family thirteen miles southwest of Seguin, and twenty-eight miles distant from our smallpox camp on York's creek. On investigation, three well developed cases of smallpox were found to exist in a negro family at the place designated. These, together with three other families who had come in contact with the infected one, were immediately quarantined. All exposed persons were vaccinated, and subsequently six other cases developed in these families, all of whom recovered and quarantine was raised on February 19th.

This makes a grand total of eighty-six cases of smallpox with three deaths having occurred in Guadalupe county from November 10, 1900, to February 19, 1901. All cases were traced to the same source of infection. All school children in the infected districts were vaccinated. About twelve hundred successful vaccinations were made by the county health officer at the expense of the county. If this had not been done, very few negroes would have been vaccinated, and in some cases it was found necessary to threaten the closing of the schools unless they would submit to it. The expense to Guadalupe county on account of this smallpox epidemic was nearly $4000, and altogether during the past year the county had to pay out on account of quarantine of infectious diseases the sum of $5000.

The disease originated in Guadalupe county from contact of the negroes with cotton pickers who had been brought here from Fort Bend county, who had suffered from that disease and had traveled here without having had their clothing changed. Although the disease is stamped out at present in Guadalupe county, it is probable that this county be infected again in the near future. The finances of the county are exhausted and it will take several years before the same can recover from the expenses which it had to meet on this account. I have no doubt that all other counties which have been afflicted similarly, and who have carried out strict quarantine measures to control the disease, are in a similar situation. The law requires a strict quarantine on infectious diseases, but provides no means to meet the expenses of the same. The counties have a general fund, raised by a tax not exceeding twenty-five cents on one hundred dollars. This is barely sufficient to meet all ordinary expenses, such as salaries of officers, expense of the courts, care of prisoners and paupers, etc., but entirely inadequate to meet extraordinary expenses, such as are incurred by epidemics. The general fund of Guadalupe county amounts to no more than $10,000 per annum, and it will readily be seen that if $5000 of this amount be taken to meet the expenses of quarantine, an amount which Guadalupe county had to expend during the past year, a deficiency is created to meet which the law makes no provsions.

The law makes it the duty of the State Health Officer to keep infectious diseases out of the Sate of Texas by proper quarantine measures, and defrays the expenses of such measures deemed necessary for that purpose. If these diseases have entered the State or developed here, it is regarded as another thing, and it is left to the different communities to deal with them as best they may. This seems entirely irrational, and I cannot understand why the State at large is not as much interested to stamp out infectious diseases within its borders as it is to keep them out. The law on quarantine should be so modified as to place the direction of quarantine matters and the combatting of infectious diseases within the State in the hands of the State Health Officer, and the State should bear the expense. This would be an enormous saving of expense. Now one county by neglect or improper measures of quarantine becomes a menace to all others, while with rigid measures adopted at once on the outbreak of any infectious disease, especially such as smallpox or yellow fever, which can be controlled by quarantine, such diseases would be short lived, and general epidemics in the State of Texas become things of the past.

DISCUSSION.

Dr. Daniel said: "I think that it is the duty of the State to take charge of the whole matter when smallpox or other disease becomes widely epidemic, especially in as much as the State undertakes to keep out diseases of foreign origin—and fails to do so, notwithstanding the whole machinery of the so-called "health department"—properly, the quarantine department—is directed to this end, and the sum total of the meagre appropriation for said department is expended in the effort. When, therefore, the disease, or any other imported disease, becomes epidemic in Texas despite the quarantine department, the State should take it in hand and pay all the expense. I feel assured that if yellow fever were to become epidemic in Texas, the State would hardly expect the particular counties where it fell with most violence to pay the cost. It would bankrupt any county in Texas were an extensive epidemic of either disease to prevail. All present remember the outbreak of smallpox among the large body of negroes returning to the States from Mexico in 1896. They were quarantined at Eagle Pass; State Health Officer Swearingen took charge. He certainly did not contemplate that Gregg county should pay the expense incident to the quarantine; and as there was no appropriation available, except the regular appropriation for keeping up the inspection stations, he was obliged to let the marine hospital service take the matter in hand. The total cost of that outbreak was a very large sum—I do not remember just how much—but several times as much as the entire appropriation for the State quarantine for all purposes. That such a thing occurred shows that it may occur again and overwhelm any county where it happens. [A bill was at the time this discussion was being held, pending in the Legislature to amend the quarantine law and Dr. Daniel discussed its features. As the bill was finally passed it threw two-thirds of the cost of local epidemics on the State and one-third on the county where it occurred; but as the Legislature made no provision for money wherewith the State should pay the two-thirds—the Governor vetoed the bill; he couldn't well do otherwise, as it would have been impracticable, had it become a law. Dr. Daniel's remarks on this head are omitted.—Ed.]

Dr. I. J. Jones, Secretary of the Quarantine Department, said, in substance, that the great difficulty in the present quarantine law for governing local epidemics is that no provision is made by which the necessary expenses can be defrayed. There is a misunderstanding on the part of some, who consider it the duty of the Governor, in case any county fails, refuses or neglects to quarantine, to order such quarantine and pay for the same. To correct this impression the opinion of the Attorney General was requested, and he decided that such was not the Governor's duty.

In order to provide a law whereby these deficiencies may be overcome, a bill was drafted investing the Governor with power when any county fails, refuses or neglects to declare quarantine, to act through the State Health Officer.

This was amended, empowering the county judge to declare quarantine through his county health officer, who shall take charge of same; one-third of the expenses to be borne by the county and two-thirds by the State, and to be administered by the State. This bill passed in the House. The Senate authorized the commissioners court to declare quarantine; one-half of the expenses to be borne by the State, and the other half by the county. In case a county fails, refuses or neglects to declare quarantine, it becomes the duty of the State Health Officer to do so at the expense of the county. In order to relieve those counties of the State that have become bankrupt from maintaining quarantines, another bill has been prepared by Senator Turney levying a tax of five cents on the one hundred dollars to be collected by the collector as other taxes are collected by each county treasurer, and to be used for no other purpose than quarantine. At the end of each year, if there is a surplus on hand, it is to be turned into the general treasury, and other taxes reduced the following year. Out of this fund the quarantine expenses shall be paid. The difficulty we meet with is in securing an adequate appropriation. Seventy-two counties reported to the State Health Officer an expenditure of $152,000. From these figures we think $300,000 would be necessary. [*Vide supra.*—Ed.]

[Note: On account of State Medical Association papers and report of the meeting, the other papers read at the meeting of health officers will have to be carried over to next month, as well as several papers from the Brazos Valley Medical Association, and the E. T. Medico-Chirurgical Society, some of which are in type.—Ed.]

